# Psychometric evaluation of the Brazilian version of the Dementia Knowledge Assessment Tool Version Two (DKAT2)

**DOI:** 10.1055/s-0046-1829130

**Published:** 2026-11-06

**Authors:** Miriane Garuzi, Hélio Amante Miot, Evelise Saia Rodolpho, Renato Bolgue Cardin, Fabio Antonio Venancio, Alessandro Ferrari Jacinto

**Affiliations:** 1 Universidade Estadual Paulista “Júlio de Mesquita Filho”, Faculdade de Medicina de Botucatu, Programa de Fisiopatologia em Clínica MédicaBotucatu SPBrazil; 2 Universidade Estadual Paulista “Júlio de Mesquita Filho”, Faculdade de Medicina de Botucatu, Programa de Pós-Graduação Profissional em Pesquisa e Desenvolvimento (Biotecnologia Médica)Botucatu SPBrazil; 3 Centro Universitário de Adamantina, Departamento de EnfermagemAdamantina SPBrazil; 4 Universidade Estadual Paulista “Júlio de Mesquita Filho”, Faculdade de Medicina de Botucatu, Departamento de Clínica MédicaBotucatu SPBrazil; 5 Centro Universitário de Adamantina, Departamento de MedicinaAdamantina SPBrazil

**Keywords:** Dementia, Caregivers, Knowledge, Psychometrics

## Abstract

**Background:**

Population aging in Brazil has increased dementia prevalence and the demand for caregivers. Caregiver knowledge is essential for care quality and clinical outcomes. Robust instruments are needed to assess dementia-related knowledge. The Dementia Knowledge Assessment Tool Version Two (DKAT2) has been transculturally adapted for Brazil.

**Objective:**

To evaluate the psychometric properties of the Brazilian version of the DKAT2.

**Methods:**

A multicenter cross-sectional psychometric study was conducted with caregivers of older adults. Content validity was assessed using the content validity index (CVI). Structural validity was analyzed through exploratory factor analysis after factorability assessment with the Kaiser-Meyer-Olkin index and Bartlett's test. Internal consistency was estimated using the Kuder-Richardson Formula 20 (KR-20). Item discrimination was examined using point-biserial and tetrachoric correlations. Temporal stability was evaluated with the intraclass correlation coefficient (ICC). Item response theory (IRT) models were used to estimate discrimination, difficulty, and guessing parameters.

**Results:**

A total of 234 caregivers were enrolled, 19 of whom completed a retest assessment. Content validity was adequate (CVI = 0.85). The internal consistency of the original 21-item instrument was low (KR-20 = 0.49). Eleven items showed poor discrimination and were excluded, resulting in a 10-item version with modest improvement (KR-20 = 0.59). Factor analysis suggested unidimensionality, although item correlations were weak. Test-retest reliability was moderate (ICC = 0.67). The IRT analysis indicated limited information across the latent trait continuum.

**Conclusion:**

The Brazilian DKAT2 showed adequate content validity and moderate temporal stability, but insufficient internal consistency and limited construct coherence. It may be used cautiously as a preliminary screening instrument, but more robust culturally-appropriate measures are needed.

## INTRODUCTION

Population aging in Brazil has intensified in recent decades, driven by lower rates of fecundity and mortality, with projections indicating that by 2050 almost 30% of the national population will comprise older adults. 1 2 This process is directly associated with a rise in the prevalence of chronic diseases, particularly dementia. This neurodegenerative condition affects more than 35 million people worldwide, roughly 10 to 20% of individuals older than 65 years, and it constitutes one of the leading causes of disability in this group. According to estimates, more than 1 million Brazilians have some type of dementia: Alzheimer's disease accounts for approximately 65% of the cases, and vascular dementia, for 30%. Progression of these conditions leads to loss of autonomy, functioning and independence, resulting in the need for continuous support, generally provided by caregivers. 3

Caregivers play a central role in this context, and their work may be formal, when there is a paid professional relationship, or informal, which is typically performed by family members, predominantly female relatives with low income and education. 4 In medium-to-high income countries, caregivers are mostly informal and face numerous challenges, including physical, emotional, and social burden. 5 The literature shows that a lack of information on dementia negatively impacts the management of symptoms, promotes worse recipient outcomes, and contributes to caregiver stress. Moreover, national studies 6 show that the main source of knowledge is still medical consultations, while less than half of caregivers seek specific informational materials, revealing a lack of formal and educational support.

In a bid to overcome these shortcomings, psychometric instruments have been developed to measure dementia-related knowledge among caregivers. 7 Although there are international scales, many have limitations in terms of validity, recency, or transcultural applicability. In this scenario, the Dementia Knowledge Assessment Tool Version Two (DKAT2) stands out. Two versions of the DKAT2 were developed in Australia, covering a broad range of clinical and prognostic aspects, besides non-pharmacological care strategies. 8 The second version, the DKAT2, was transculturally adapted for Brazil in 2017, but it has yet to be validated in the country, a fundamental stage to ensure its reliability and relevance in planning public health strategies aimed at caring for older adults with dementia. 9

The use of a questionnaire with comprehensive, robust content and plain language is paramount to better assess the knowledge held by caregivers, informing the development of educational strategies in public health based on the deficits identified, as well to analyze the impact of these strategies during follow-up.

Recent studies 10 emphasize that dementia knowledge is a multidimensional construct, encompassing biomedical understanding, care practices, risk perception, and social attitudes. Instruments that fail to account for this multidimensionality may exhibit reduced internal consistency and limited construct validity, particularly across diverse sociocultural contexts.

Although the DKAT2 has been applied in different cultural contexts, psychometric properties may vary according to linguistic, cultural, and educational factors. Transcultural adaptation alone does not ensure that an instrument will demonstrate adequate reliability and validity in a new population. Therefore, it is essential to evaluate the psychometric performance of such instruments in the populations in which they are intended to be used.

The present study aimed to evaluate the psychometric properties of the Brazilian version of the DKAT2 among caregivers of older adults.

## METHODS

### Study design and ethical aspects

The current was a multicenter, cross-sectional, psychometric study conducted in Brazil. The study was approved by the Ethics in Research Committee of Faculdade de Medicina de Botucatu – Universidade Estadual Paulista “Júlio de Mesquita Filho” (UNESP; approval number: 3.320.895). All participants provided written informed consent before data collection.

### Participants

A convenience sample of caregivers of older adults with dementia was recruited between December 2018 and December 2022. The participants were enrolled from multiple settings, including primary care units, hospital inpatient and emergency services, long-term care facilities, community centers, and outpatient clinics. Eligible participants were adults (aged ≥ 18 years) who provided care to older adults diagnosed with dementia, either as formal (paid professionals) or informal (family members or unpaid individuals) caregivers. No exclusion criteria were applied regarding the level of schooling or the duration of the caregiving, to reflect real-world heterogeneity.

The sample size was estimated based on psychometric recommendations of at least 10 participants per item for exploratory analyses of instruments with binary responses, resulting in a minimum target of approximately 210 participants. This approach is consistent with recommendations for factor analysis and item response modeling in preliminary validation studies. 11 Given the multicenter design and feasibility constraints, a subsample of participants was invited to fill out the questionnaire twice for test-retest analysis.

### Evaluation protocol

Data were collected using a structured questionnaire administered either in person (paper format) or online (Google Forms, Alphabet Inc.). The instrument included:

- Sociodemographic variables: age, sex, level of schooling, type of caregiver (formal/informal), and relationship to the care recipient; and
- the Brazilian version of the DKAT2 ( Table 1 ). 9

**Table 1:** Adapted Dementia Knowledge Assessment Tool Version Two (DKAT2)

Here are some statements about dementia. Please read each statement carefully and place a tick in the box to show if you agree or disagree with the statement, or if you do not know. It is important to tick only one box (“yes,” “no,” or “I do not know”) for every statement.				
Statements about dementia	Yes	No	I do not know
1. Dementia occurs because of changes in the brain.	1		
2. Brain changes causing dementia are often progressive.	1		
3. Alzheimer's disease is the main cause of dementia.	1		
4. Blood vessel disease can also cause dementia.	1		
5. Confusion in an older person is almost always due to dementia.		1	
6. Only older adults develop dementia.		1	
7. Knowing the likely cause of dementia can help to predict its progression.	1		
8. Incontinence always occurs in the early stages of dementia.		1	
9. Dementia is likely to limit life expectancy.	1		
10. When a person has late-stage dementia, families can help others to understand that person's needs.	1		
11. People who have dementia may develop problems with visual perception (understanding or recognizing what they see).	1		
12. Sudden increases in confusion are characteristic of dementia.		1	
13. Uncharacteristic distressing behaviors may occur in people who have dementia (e.g., aggressive behavior in a gentle person).	1		
14. Difficulty swallowing occurs in late-stage dementia.	1		
15. Movement (e.g., walking, moving in a bed or chair) is limited in late-stage dementia.	1		
16. Changing the environment (e.g., putting on a CD, opening or closing the blinds) will make no difference to a person who has dementia.		1	
17. When a person who has dementia is distressed, it may help to talk to them about their feelings.	1		
18. It is important to always correct a person who has dementia when they are confused.		1	
19. A person who has dementia can often be supported to make choices (e.g., what clothes to wear).	1		
20. It is impossible to tell if a person who is in the later stages of dementia is in pain.		1	
21. Exercise can sometimes be of benefit to people who have dementia.	1		

Note: Adapted from Piovezan et al. 9 (2018).

The participants completed the questionnaire independently. During the in-person administration, clarification of terms was allowed when requested, to ensure comprehension. For online responses, no direct assistance was available. The DKAT2 is composed of dichotomous items (correct/incorrect) designed to assess knowledge on dementia across clinical, prognostic, and care-related domains.

### Psychometric framework

The psychometric evaluation followed contemporary validity theory, in which validity is considered a unified construct supported by multiple sources of evidence, including content validity, internal structure, and relationships with other variables. Internal structure was examined through complementary approaches derived from classical test theory and item response theory (IRT), enabling a comprehensive assessment of item behavior and scale coherence. 12

Content validity indicates whether the items adequately represent the domain intended to be measured, and it was established by 10 healthcare professionals involved in treating older adult patients with dementia. The experts classified the relevance and representativeness of the items contained in the instrument. The content validity index (CVI) was determined by adding the pertinent answers and dividing the result by the number of experts.

Structural validity was estimated using exploratory factor analysis (EFA). 13 Initially, the factorability of the questionnaire was examined by the Kaiser-Meyer-Olkin (KMO) method and Bartlett's test of sphericity. 14 The EFA was performed using a tetrachoric correlation matrix. Factors were extracted using principal axis factoring (PAF), which is recommended for dichotomous variables and latent trait estimation. Because the retained solution was unidimensional, no factor rotation was applied.

Dimensionality refers to the extent to which the items belong to a single underlying construct within an instrument; it was assessed using Horn's parallel analysis, confirmatory factor analysis (CFA), and visual analysis of the scree plot. 15 16 Despite this, statistical unidimensionality does not necessarily imply conceptual coherence or satisfactory reliability. Internal consistency reflects the extent to which items behave coherently as indicators of the same construct. As the DKAT2 items are dichotomous, internal consistency was estimated using the Kuder-Richardson Formula 20 (KR-20). Item-total discrimination was examined through point-biserial correlations. Test-retest reliability, temporal stability, or whether scores remain reasonably consistent over time when the construct is expected to be stable was assessed with the intraclass correlation coefficient (ICC), since values ≥ 0.60 were considered indicative of moderate agreement. Finally, IRT was applied to examine item discrimination, difficulty, and guessing parameters, enabling the identification of poorly-informative items. 17 18

### Item response theory analysis

The IRT was applied to examine item-level performance. Item IRT analyses were conducted using a two-step approach. Initially, item discrimination parameters were estimated to identify poorly-informative items (a < 0.5). Subsequently, a three-parameter logistic model (3PL) was applied to evaluate item discrimination, difficulty, and guessing behavior. This approach enabled a more nuanced assessment of item performance beyond classical test theory metrics. 11 The 3PL model was selected because the DKAT2 consists of dichotomously-scored items, and it includes response options that may be influenced by guessing or uncertainty, particularly among participants with limited dementia-related knowledge. The inclusion of the guessing parameter enables the model to account for the possibility that some correct responses may occur independently of the respondent's underlying level of knowledge.

### Statistical analysis

All analyses were conducted using the R software (free and open source), version 4.2.1, with the packages *psych* and *mirt* . Descriptive statistics were used to characterize the sample and item response distribution. Psychometric analyses followed a sequential approach, including content validity, internal structure, reliability, and item response modeling.

## RESULTS

A total of 234 caregivers of older adults participated in the study, including 19 who completed a retest assessment. Psychometric analyses were performed using all available questionnaire administrations ( *n* = 253), whereas descriptive item frequencies were calculated using only the first administration to each participant ( *n* = 234).

Of all questionnaire administrations, 181 were conducted in person and 72, online. The sample was predominantly composed of female (85%) and informal (60%) caregivers, with just over half of the informal caregivers being children of the care recipient. The participants had a mean age of 48 (range: 18–83) years. Approximately half of the sample had completed secondary education; however, schooling data were missing for 33% of the participants, predominantly among those who filled out the questionnaire online.

For the DKAT2, the mean number of correct responses was 14 ± 2, with scores ranging from 7 to 20. Items 1, 2, 6, 7, 10, 11, 13, 15, and 21 were answered correctly by more than 80% of the participants. In contrast, items 5 and 12 showed incorrect response rates exceeding 50%. Overall, the “I do not know” option accounted for 11% of all responses, with items 3, 4, 8, and 20 exhibiting the highest frequencies of this response category ( Table 2 ).

**Table 2:** Frequency distribution of responses ( *n* = 234)

Item	Correct	Incorrect	I do not know
	N (%)	N (%)	N (%)
1	214 (91.5)	8 (3.4)	12 (5.1)
2	206 (88)	11 (4.7)	17 (7.3)
3	107 (45.7)	81 (34.6)	46 (19.7)
4	138 (59)	33 (14.1)	63 (26.9)
5	77 (32.9)	135 (57.7)	22 (9.4)
6	197 (84.2)	22 (9.4)	15 (6.4)
7	218 (93.2)	8 (3.4)	8 (3.4)
8	97 (41.5)	83 (35.5)	54 (23.1)
9	149 (63.7)	61 (26.1)	24 (10.3)
10	224 (95.7)	4 (1.7)	6 (2.6)
11	205 (87.6)	13 (5.6)	16 (6.8)
12	40 (17.1)	167 (71.4)	27 (11.5)
13	209 (89.3)	11 (4.7)	14 (6)
14	164 (70.1)	33 (14.1)	37 (15.8)
15	191 (81.6)	20 (8.5)	23 (9.8)
16	143 (61.1)	73 (31.2)	18 (7.7)
17	174 (74.4)	34 (14.5)	26 (11.1)
18	105 (44.9)	102 (43.6)	27 (11.5)
19	182 (77.8)	38 (16.2)	14 (6)
20	109 (46.6)	78 (33.3)	47 (20.1)
21	212 (90.6)	11 (4.7)	11 (4.7)

During the in-person administration, several caregivers requested clarification regarding the meaning of the term *dementia* . Questionnaire completion time was recorded for 9 participants, and it ranged from 2 to 33 minutes, with a mean duration of 12 minutes, which was considered acceptable for a knowledge assessment instrument.

The individual item CVI proved adequate for most items, except for items 1, 5, 6, 17, and 18. The overall CVI of 0.85 was considered satisfactory.

The original 21-item DKAT2 demonstrated low internal consistency (KR-20 = 0.49), indicating limited coherence among items. Item-level analyses indicated that more than half of the items showed suboptimal psychometric properties, including low discrimination (a < 0.5), and/or elevated pseudoguessing (g > 0.5), and limited information across the latent trait continuum. Consequently, these items demonstrated a reduced capacity to differentiate respondents according to their level of dementia-related knowledge, as evident on visual analysis of the item information curves ( Figure 1 and **Supplementary Material Table S1** – available at https://www.arquivosdeneuropsiquiatria.org/wp-content/uploads/2026/07/ANP-2025.0414-Supplementary-Material.docx ).

**Figure 1:**
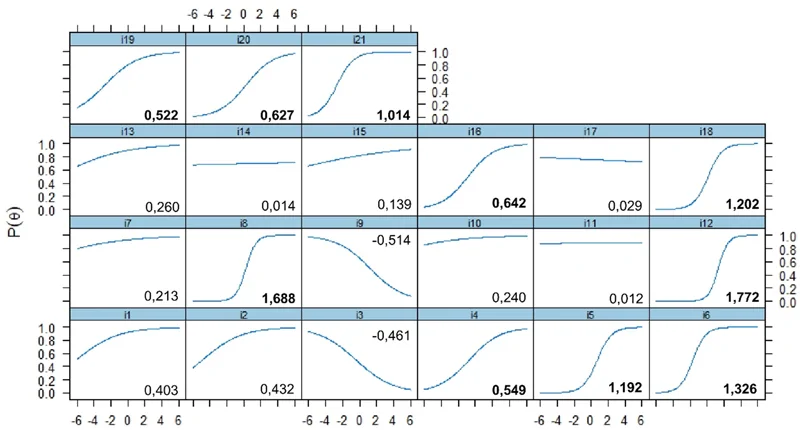
Characteristic curve of items from the Dementia Knowledge Assessment Tool Version Two (DKAT2; *n* = 253).

The reduced 10-item questionnaire was evaluated using EFA based on a tetrachoric correlation matrix. The KMO index of 0.72) and Bartlett's test of sphericity (Chi-squared [X ^2^ ] = 174.5; degrees of freedom [df] = 45; *p* < 0.001) supported sampling adequacy. The first factor explained 26% of the variance, and the ratio between the first and second eigenvalues was 2.6. Horn's parallel analysis supported a unidimensional structure, which was also consistent with visual inspection of the scree plot, showing a clear elbow after the first factor ( Figure 2 ). The one-factor CFA model, estimated using weighted least squares means and variance adjusted (WLSMV), demonstrated adequate fit (χ ^2^ = 34.4; df = 45; *p* > 0.05; CFI (Comparative Fit Index) = 0.99; TLI (Tucker–Lewis index) = 0.99; RMSEA (root mean square error of approximation) = 0.01, 95%CI: 0.00–0.03), providing additional support for a unidimensional structure. Internal consistency was suboptimal, with a KR-20 (Kuder-Richardson coefficient) coefficient of 0.59 (95%CI: 0.51–0.66).

**Figure 2:**
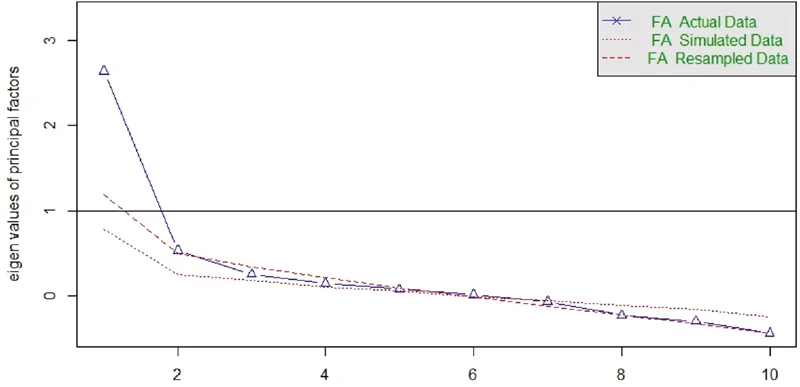
Exploratory factor analysis with scree plot after reducing items.

The analysis of tetrachoric inter-item correlations revealed that several item pairs showed weak associations, with correlation coefficients below the commonly-accepted threshold of 0.30. Point-biserial item-total correlations identified a subset of items with satisfactory discrimination, demonstrating significant correlations with the total score ( *p* < 0.05) and coefficients exceeding 0.30.

According to the IRT 3PL ( Table 3 ), the retained items showed predominantly moderate to high discrimination parameters (a = 0.52–2.45), indicating an adequate ability to differentiate respondents according to dementia-related knowledge. The item difficulty parameters ranged from -2.72 to 1.34, suggesting coverage of low to moderately-high levels of the latent trait. Most items exhibited negligible guessing parameters, although items 4 and 20 showed elevated pseudoguessing estimates. Overall, the item parameter estimates indicate that the reduced version performs best in distinguishing respondents with low-to-moderate levels of knowledge, while providing less information at the upper end of the latent trait continuum.

**Table 3:** Discrimination (a), difficulty (b), guessing (g), RMSEA, and S-X ^2^ statistic parameters derived from the item response theory analysis of the reduced 10-item DKAT2 ( *n* = 253)

Items	Discrimination (SE)	Difficulty (SE)	Guessing (SE)	RMSEA	S-X ^2^	*p* -value
4	1.76 (1.57)	-2.72 (0.46)	0.46 (0.17)	0.07	10.94	0.05
5	1.16 (0.26)	-2.67 (0.18)	0.00 (0.00)	0.00	1.78	0.78
6	1.49 (0.44)	-1.56 (0.30)	0.00 (0.02)	0.00	1.51	0.68
8	1.86 (0.90)	-0.93 (0.25)	0.05 (0.14)	0.00	2.42	0.49
12	1.76 (0.52)	0.36 (0.26)	0.00 (0.00)	0.05	4.74	0.19
16	0.58 (0.21)	0.57 (0.39)	0.00 (0.03)	0.00	3.59	0.61
18	2.45 (1.83)	0.73 (0.46)	0.22 (0.25)	0.06	5.83	0.12
19	0.52 (0.24)	0.96 (1.20)	0.01 (0.15)	0.00	2.75	0.74
20	1.98 (1.63)	1.10 (0.27)	0.32 (0.10)	0.07	10.66	0.06
21	0.98 (0.33)	1.34 (0.73)	0.00 (0.06)	0.05	6.52	0.16

Abbreviations: DKAT2, Dementia Knowledge Assessment Tool Version Two; SE = standard error; RMSEA = root mean square error of approximation; S-X2 = S-X2 statistic

The comparison between the two-parameter logistic model (2PL) and the 3PL models showed no significant improvement in fit (ΔX ^2^ = 3.55; df = 10; *p* = 0.966), but the 3PL model was retained for exploratory assessment of pseudoguessing and because item-level parameter interpretation remained stable across models.

The fit of the 1-factor 3PL IRT model was satisfactory (M2 = 14.99; df = 25; *p* = 0.94), with excellent approximate fit indices (RMSEA = 0.00; 95%CI: 0.00–0.01; SRMSR = 0.044; CFI = 0.99; TLI = 0.99), supporting the adequacy of a unidimensional structure. The item-level fit statistics indicated adequate fit of all retained items to the unidimensional IRT model. No item showed significant misfit according to the summed-score (S) and Pearson X ^2^ (S-X ^2^ ) statistic (all *p* > 0.05), and item-level RMSEA values were uniformly low (< 0.08). These findings suggest that the retained items were generally consistent with the proposed latent structure, although the overall strength of the common dimension remained limited, as reflected by the modest reliability and explained variance estimates.

Item information curves indicated that items 16 and 19 contributed relatively little information across the latent trait continuum, whereas the remaining items exhibited distinct information peaks, suggesting adequate discrimination, but only within a limited range of ability (Theta, θ) ( Figure 3 ).

**Figure 3:**
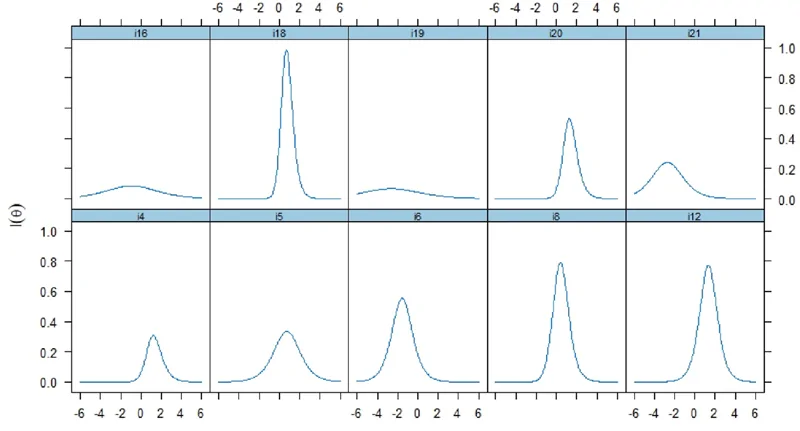
Item information curves.

Temporal stability was assessed using a test-retest procedure in a subsample of 19 caregivers. The number of correct responses ranged from 3 to 10 in the initial assessment, and from 4 to 9 in the retest. The mean scores were similar across both administrations, averaging approximately six correct responses. The ICC for absolute agreement was 0.67 (95%CI: 0.37–0.87), indicating moderate temporal stability.

## DISCUSSION

In the present study, we evaluated the psychometric properties of the Brazilian version of the DKAT2 and identified important limitations in its internal structure. Although the instrument demonstrated adequate content validity and acceptable temporal stability, its internal consistency remained below recommended thresholds, even after item reduction. This finding indicates that the items do not behave as coherent indicators of a single construct, limiting the reliability of the total score. 13 14

The low internal consistency observed suggests that the DKAT2 may not adequately represent a unidimensional construct of dementia-related knowledge. Although the EFA suggested a single dimension, this result should be interpreted with caution, as statistical unidimensionality does not necessarily imply conceptual coherence. Dementia knowledge has been increasingly described as a multidimensional construct, encompassing biomedical aspects, caregiving practices, disease trajectory, and psychosocial understanding. 10 Instruments that aggregate these domains into a single score may show reduced internal consistency, particularly in populations with heterogeneous schooling and sociocultural backgrounds.

The apparent discrepancy between unidimensionality and low internal consistency is not uncommon in psychometric studies. While dimensionality reflects the presence of a common latent construct, reliability reflects the strength of the relationships among items. Thus, the identification of a single factor does not necessarily imply adequate internal consistency. In the present study, the low KR-20 suggests that the latent dimension identified was relatively weak, and that the items did not converge strongly toward a homogeneous construct. Consistent with this interpretation, although Horn's parallel analysis and the CFA supported the retention of a single factor, the modest ratio between the first and second eigenvalues (2.6) and the limited variance explained by the first factor (26%) indicate that the dominant dimension only accounted for a small proportion of the overall item variance.

The findings from the IRT analysis reinforce this interpretation. A substantial proportion of items showed low discrimination, indicating limited ability to differentiate individuals according to their level of knowledge. In addition, the item information curves revealed that most items contribute information only within a narrow range of ability, reducing the instrument's sensitivity across the full spectrum of caregiver knowledge. These characteristics limit its usefulness for clinical assessment and research applications. 11

Compared with validation studies conducted in other countries, such as Germany, 7 Spain, 19 and Greece, 20 the Brazilian version showed inferior psychometric performance. While sociocultural and linguistic factors may partially explain these differences, the present results suggest that such factors alone are insufficient. Instead, the findings point to intrinsic limitations to the structure of the instrument, including item heterogeneity and possible mismatch between item complexity and the cognitive demands of the target population.

Meanwhile, the assessment of knowledge on dementia is still underdefined. In Australia, 21 head-to-head psychometric comparisons demonstrate that widely-used dementia-knowledge instruments, including the DKAT2, the Alzheimer's Disease Knowledge Test (ADKT), the Alzheimer's Disease Knowledge Scale (ADKS), and the Dementia Knowledge Twenty (DK-20), present only marginal reliability and inconsistent convergence, highlighting the inherent difficulty in capturing dementia knowledge as a single, unified construct.

Health literacy is a relevant factor in this context. Recent studies 22 demonstrate that caregiver health literacy directly influences understanding of dementia, decision-making, and care outcomes. In addition, limitations in digital and functional health literacy have been shown 23 to reduce caregivers' ability to access, interpret, and apply health information effectively. In the present study, the frequent need for clarification of basic terms during the in-person administration supports the hypothesis that comprehension difficulties may have contributed to inconsistent responses and reduced item discrimination. The frequent need for clarification of the term *dementia* during questionnaire administration suggests that linguistic comprehension and health literacy may have influenced response patterns, potentially contributing to lower item discrimination and affecting the construct validity of the instrument.

From a practical perspective, the DKAT2 may still be useful as a brief screening instrument, particularly to identify caregivers with lower levels of knowledge. The reduced 10-item version showed modest improvement in psychometric performance, and it may be useful in settings requiring rapid assessment. However, its internal consistency remains suboptimal, which limits its application as a standalone measure, especially in studies evaluating educational interventions or longitudinal outcomes. Although item retention was primarily guided by psychometric performance, content coverage was also reviewed to ensure representation of different dementia-related knowledge domains. Nevertheless, future studies should combine psychometric and content-validity criteria during item selection.

Although the DKAT2 has demonstrated ease of application and usefulness as a screening instrument, it has some major methodological and psychometric limitations. These shortcomings include an overemphasis on biomedical domains, a risk of ceiling effects in highly-educated populations, and the presence of ambiguous items. The original authors 8 acknowledge the need for more comprehensive, culturally-sensitive versions covering biomedical, practical, and psychosocial dimensions of care. Therefore, the results of the present study highlight the importance of developing more robust instruments to assess dementia-related knowledge that are adapted to the sociocultural particularities of the Brazilian population.

These findings also have broader implications for the assessment of dementia-related knowledge. Recent evidence 24 highlights the importance of structured dementia-literacy interventions in improving caregiver outcomes, reinforcing that knowledge is not a unitary construct, but one that involves multiple domains that may require distinct measurement strategies. Therefore, future instruments should consider multidimensional frameworks, clearer language, and cultural adaptation aligned with the health-literacy profile of the target population.

## Study limitations

The current study has limitations that should be considered. First, the use of convenience sampling and the heterogeneity of the participants (formal and informal caregivers) may have influenced the results. These groups differ in terms of experience, training, and exposure to dementia-related information, which may affect response patterns and psychometric performance. In addition, a substantial proportion of missing data regarding the level of schooling limited the ability to perform subgroup analyses and to directly assess the influence of education and health literacy.

Second, the use of different types of data collection (in-person and online) may have affected comprehension and response behavior. While the in-person administration enabled the clarification of doubts, online completion may have increased the misunderstanding of items and contributed to missing data. The relatively-small retest subsample also limits the precision of temporal-stability estimates, although it was considered acceptable for a preliminary assessment in a multicenter feasibility context.

The pattern of missing data also warrants consideration. Missing information on the level of schooling occurred predominantly among participants who completed the questionnaire online, suggesting that the missingness may not have been completely at random. Although the available data did not enable a formal assessment of missing-data mechanisms, the possibility that missingness was related to participant characteristics or the type of administration cannot be excluded. Consequently, some degree of bias may have been introduced into subgroup descriptions and the interpretation of factors associated with questionnaire performance.

Third, important measurement limitations were identified. Although the ICC suggested moderate temporal stability, the associated 95%CI was wide, indicating limited precision of the estimate. In addition, the absence of qualitative approaches, such as cognitive interviewing, limited the understanding of how participants interpreted the items. Future studies with larger samples and more comprehensive psychometric approaches, including multidimensional modeling, are needed to confirm these findings.

In conclusion, the Brazilian DKAT2 showed acceptable content validity and temporal stability, but insufficient internal consistency and limited item discrimination. Therefore, although it may retain some usefulness as a preliminary screening questionnaire, the present findings do not support its broad use as a psychometrically-robust standalone measure of dementia-related knowledge in Brazilian caregivers. Future instruments should use clearer wording, stronger cultural adaptation, a better balance of item difficulty, and broader coverage of the biomedical, practical, and psychosocial aspects of dementia care.
